# The role of inflammation in immune system of diabetic retinopathy: Molecular mechanisms, pathogenetic role and therapeutic implications

**DOI:** 10.3389/fimmu.2022.1055087

**Published:** 2022-12-13

**Authors:** Tong Yue, Yu Shi, Sihui Luo, Jianping Weng, Yali Wu, Xueying Zheng

**Affiliations:** ^1^ Department of Endocrinology, the First Affiliated Hospital of USTC, Division of Life Sciences and Medicine, University of Science and Technology of China, Hefei, Anhui, China; ^2^ Department of Ophthalmology, the First Affiliated Hospital of USTC, Division of Life Sciences and Medicine, University of Science and Technology of China, Hefei, Anhui, China

**Keywords:** diabetic retinopathy, immune inflammation, therapy, cytokines, chemokines

## Abstract

Diabetic retinopathy is one of the most common complications of diabetes mellitus and the leading cause of low vision and blindness worldwide. Mounting evidence demonstrates that inflammation is a key mechanism driving diabetes-associated retinal disturbance, yet the pathophysiological process and molecular mechanisms of inflammation underlying diabetic retinopathy are not fully understood. Cytokines, chemokines, and adhesion molecules interact with each other to form a complex molecular network that propagates the inflammatory and pathological cascade of diabetic retinopathy. Therefore, it is important to understand and elucidate inflammation-related mechanisms behind diabetic retinopathy progression. Here, we review the current understanding of the pathology and pathogenesis of inflammation in diabetic retinopathy. In addition, we also summarize the relevant clinical trials to further suggest inflammation-targeted therapeutics for prevention and management of diabetic retinopathy.

## Introduction

Diabetic retinopathy (DR) is a common microvascular complication of type 1 and type 2 diabetes. DR is the leading cause of low vision and blindness in patients with diabetes and can severely affect people of all ages worldwide, with a prevalence of 34.6% (93 million) in adults aged 40 years and over (1). A systematic review focused on population-based studies estimated that DR has an annual incidence ranging from 2.2% to 12.7% (2). Epidemiological evidence suggests that DR not only increases the risk of vision impairment and blindness in diabetic patients but also increases the risk of all-cause and cardiovascular disease (CVD) mortality in a multi-ethnic Asian population (3, 4).

DR is classified as nonproliferative diabetic retinopathy (NPDR) and proliferative diabetic retinopathy (PDR) according to the modified Airlie House Classification used in the Early Treatment Diabetic Retinopathy Study (ETDRS) (5). The earliest morphological sign of NPDR is the formation of microaneurysms, in which the capillary wall expands outwards, detected by ophthalmoscopy with blot hemorrhages (6). Further signs of NPDR are changes in retinal blood flow and vascular permeability, thickening of the basement membrane, loss of pericytes, and formation of the acellular capillary. As the severity of ischemia increases, it may develop into PDR. PDR is characterized by the hallmark feature of pathologic retinal neovascularization, with vitreous hemorrhage, vitreous new blood vessels, and retinal traction detachment, which will lead to blindness (7).

Diabetic macular edema (DME) is an important additional classification in DR that is associated with ischemia because of the increased permeability of retinal capillaries and microaneurysms, resulting in extracellular fluid accumulation and normal dense macular tissue thickening. DME can arise at any stage of NPDR and PDR and threatens visual acuity (8). Overall, T1DM patients tended to develop diabetic retinopathy and PDR, while T2DM patients treated with insulin were more likely to develop DME.

The pathophysiology of DR is driven by the interaction of many factors, among which long-term episodes of hyperglycemia (elevated blood glucose levels) are an important factor in diabetic patients (9). In DR patients, elevated blood glucose levels lead to abnormal regulation of many biochemical pathways, hyperglycemia-induced increases in the flux of advanced glycation end products/receptors (AGE/RAGE), the polyol pathway, protein kinase C (PKC) activation, and the hexosamine pathway. These modifications also result in mitochondrial failure, inflammation, and hypoxia-driven vascular endothelial growth factor (VEGF) secretion, leading to vascular and neuronal apoptosis, neovascularization, and vascular permeability, respectively (10, 11). The complex aetiology of DR reflects the various treatments currently available, including laser photocoagulation, glucocorticoids, vitrectomy, and drugs that neutralize VEGF. Due to painful patient administration and long-term adverse effects, the use of argon laser and intravitreal injection therapeutic approaches is limited (12). There is interest in developing pharmacological therapies for DR management conditions, such as nonsteroidal anti-inflammatory drugs (NSAIDs) that inhibit or delete proinflammatory molecules, anti-VEGF agents, and antitumour necrosis factor α (TNF-α) agents (13). Therefore, a deeper comprehension of fundamental processes and innovative treatments in DR is needed.

In this review, we concentrate on the involvement of inflammation in the pathophysiology of DR and summarize the recent advances in current and emerging treatments for DR.

## Inflammation in diabetes and diabetic retinopathy

For many years, it has been proposed that chronic tissue inflammation may play a role in metabolic illness. Chronic inflammatory problems in the peripheral and central nervous systems are caused by diabetes. A substantial amount of evidence from both patients and animal models demonstrates that DR is a chronic low-grade inflammatory illness involving inflammatory mediators.

### Inflammation in diabetes

Type 1 diabetes (T1D) is a condition caused by autoimmune damage or loss of functional β cell mass. Low insulin secretion capability, self-antigen presentation, and immune-mediated destruction are hypothesized to be the results of cytokine-driven inflammation and other stress factors (14). Both CD4+ and CD8+ T cells are involved in the development of T1D and play a role in the different stages of T1D to promote the destruction of pancreatic β cells and the pathogenesis of the disease (15). These adaptive immune cells regulate the inflammatory response and destroy insulin-producing β cells by secreting proinflammatory cytokines such as TNF-α, interferon γ (IFN-γ), and interleukin-1 (IL-1) (16).

In addition to causing oxidative stress, oxygen species (ROS) can stimulate the growth of macrophages and dendritic cells by activating the nuclear factor kappa light chain enhancer (NF-κb) pathway, activator protein-1 (AP-1), and mitogen-activated protein kinase (MAPK) (17). Type I interferon (IFN) is a cytokine essential for innate and adaptive immune responses. Levels of type I interferon as well as mediated signaling were also found to be upregulated in children at high risk for T1D and in new-onset T1D patients (18). In β cells, activation of type I IFN signaling leads to high expression of major histocompatibility complex (MHC) class I, epigenetic changes, endoplasmic reticulum (ER) stress, and induction of post-transcription and post-translation modifications (19). This may result in the persistent presentation of neoantigens to the immune system and apoptosis of β cells. Innate immunity and inflammatory mediators can impact T1D, contribute to destroying pancreatic β cells and cause peripheral insulin resistance (20). The study of blocking inflammatory factors such as the IFN inhibitor golimumab in youth with new-onset T1D has achieved some success (21).

The pathogenesis of type 2 diabetes (T2D) is closely related to obesity and insulin resistance, which leads to a burden on beta cells, which eventually depletes them, leading to hyperglycemia (22). Studies have shown that people with prediabetes have an increase in several inflammatory markers, such as resistin, interleukin 6 (IL-6), TNF-α, interleukin 1β (IL-1β), and monocyte chemoattractant protein-1 (MCP-1), in their serum and fasting glucose levels (23). As the adipose tissue increases and as various metabolic pressures build up, the cytokines released by the adipose tissue “Spill over”, which can create an imbalance between cytokines that promote insulin sensitivity, including adiponectin, leptin, and proinflammatory cytokines (24). T2D frequently exhibits low-grade inflammation, and the maturation of local macrophages is crucial in controlling this process. T2D-related inflammation is characterized by an increase in macrophages in different tissues and the simultaneous production of TNF-α, IL-6, IL-1β, and interleukin 8 (IL-8) cytokines (25). The underlying mechanism of insulin resistance is also related to the inflammatory response, with activation of inflammasomes in islet inflammatory cells impairing islet function and viability. Additionally, it appears that the development of diabetic ophthalmological problems involves an inflammatory process. Inflammation is a nonspecific response to injury or stress, including various functional and molecular mediators, leukocyte recruitment, and/or activation (26). An acute inflammatory response can eliminate infectious agents, but if it persists for a long time, it may have an adverse effect.

Inflammation, immune cell modulation, survival, and proliferation are all impacted by toll-like receptors (TLRs), which are the first line of defense against pathogen invasion and identify various pathogen-associated chemical patterns (27). The hallmark of chronic inflammation is tissue filled with macrophages, lymphocytes, and mature B cells. As a result of the long-term release of inflammatory factors in the tissue, these white blood cells continue to exude from the blood vessels and eventually accumulate in the tissue (28). Inflammatory dysregulation can also lead to tissue and organ damage, which promotes disease. Inflammation is involved in the development of DR, so understanding the inflammatory process may provide new strategies for DR therapy.

### Inflammation in DR

**Figure 1:**
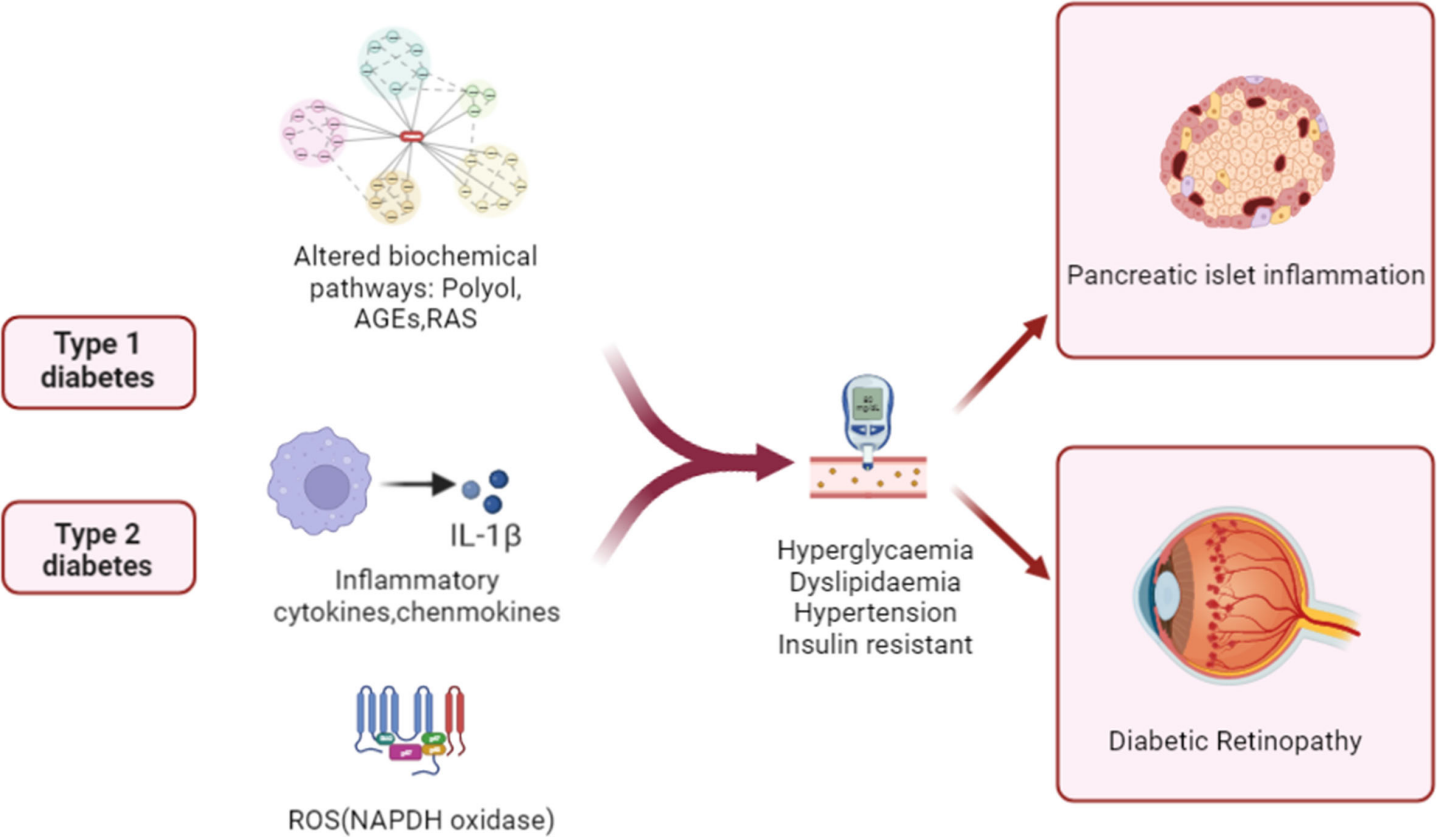
Schematic illustration of pathogenic mechanisms leading to pancreatic β cell damage and sight-threatening endpoints of diabetic retinopathy (DR). In patients with diabetes, inflammation, aberrant signaling of trophic factors, and biochemical pathways are upregulated. The alterations then enter the systemic circulation and contribute to diabetic pathology and islet inflammation by increasing levels of blood glucose and lipids and insulin resistance.

The pathophysiology of DR is complicated, and the disease’s fundamental processes are not fully understood. **Figure 1** summarizes the key diabetes-related factors associated with the development of T1D, T2D, and DR. Elevated intracellular glucose levels in diabetic individuals trigger the polyol pathway, which metabolizes glucose (29). This leads to the deposition of AGEs, the activation of PKC, and the upregulation of AGE receptors and the hexokinase pathway (30). It triggers oxidative stress, which causes a rise in intracellular reactive oxygen species (ROS) and irreparable cellular damage. A hyperglycemic environment leads to metabolic dysfunction, oxidative stress, and the production of ROS, such as superoxide radicals (31). Apoptosis may be caused by mitochondrial abnormalities, hypoxia-mediated VEGF production may be increased by inflammatory stimuli, and VEGF is a crucial angiogenesis mediator (32). Studies have shown that circulating mitochondrial DNA (mtDNA) levels are associated with diabetic retinopathy, and high blood glucose-induced mtDNA changes in early diabetes may contribute to inflammation and diabetic retinopathy progression (33). Chronic inflammation can result from chronic hyperglycemia and oxidative stress, as well as other molecular mediators. Increased levels of chemokines, including MCP-1, CCL2 and CCL5, as well as proinflammatory cytokines, such as TNF-α, IL-1β, and IL-6, were found in DR (34). Activated cytokines secrete intracellular adhesion molecules, such as ICAM-1 and VCAM-1, which attract monocytes and leukocytes and promote a continuous inflammatory response (35). With the accumulation of chronic inflammation, inflammatory cells infiltrate and destroy tissues, further aggravating retinal vascular permeability, vasodilation, and retinal thickening in DR patients.

**Figure 2:**
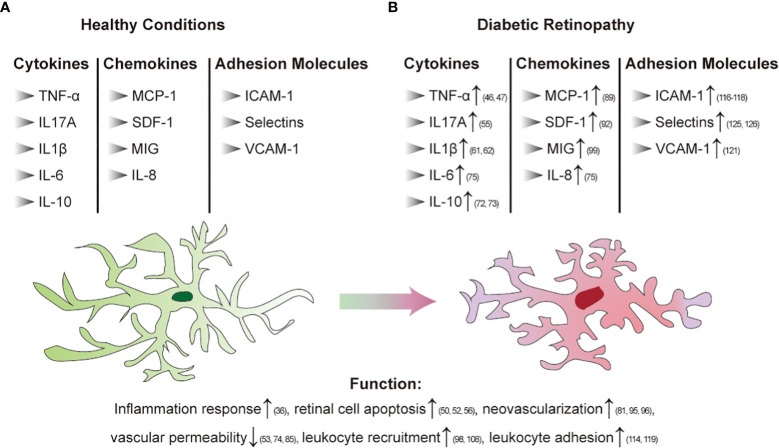
Immune regulation in diabetic retinopathy [**(A)**: Healthy conditions; **(B)** DR]. Arrows indicate elevated levels or increased activity.

Inflammation plays an important role in the pathogenesis of DR. In animal models and patients with diabetes, chronic low-grade inflammation is widely found at different stages of DR (36). It has been established that leukocytosis is a crucial step in the early stages of DR and is related to adhesion molecule-mediated leucocyte-endothelial adhesion. PDR vitreous bodies also cause proinflammatory activation of endothelial cells. The nuclear translocation of the proinflammatory transcription factors NF-κB and pCREB, ROS production, disruption of endothelial barrier integrity, E-selectin, the upregulation of VCAM-1 and ICAM-1 and the increase in leukocyte adhesion were observed. Studies have found that both soluble E-selectin and SVCAM-1 levels are elevated in diabetic retinopathy patients and that CCL17, CCL19, and TGF β are significantly upregulated (37, 38). It has been reported that chemokines that regulate the attraction and activation of leukocytes have a role in the development of DR. Patients with DR had higher levels of chemokines such as MCP-1, macrophage inflammatory protein-1 alpha (MIP-1 α) and MIP-1 beta (39). In addition, retinal neuroglia dysfunction is also associated with the development and expansion of retinal inflammation in DR (40). In general, chronic inflammation in diabetes leads to the response of inflammatory cells in the body, which further affects capillary dysfunction and ultimately leads to DR. Therefore, inflammation as a fundamental cause of DR still needs further understanding to solve this problem. **Figures 2A, B** summarize the changes caused by chronic inflammation of DR.

### Inflammatory cytokines

Cytokines are a wide range of molecular families with different structures and individual proteins known for their many roles in the immune system. Some cytokines mediate downstream responses through the JAK/STAT signaling pathway, such as IL-6, and others activate the NF-κB signaling pathway, such as IL-1 and IL-17 (41). Some cytokines have a clear role in promoting inflammation, such as IL-1 and TNF, known as proinflammatory cytokines, and some cytokines, such as IL-4 and IL-10, suppress proinflammatory activity, called anti-inflammatory cytokines. Proinflammatory cytokines upregulate the expression of proinflammatory genes encoding enzymes that synthesize leukotrienes, platelet-activating factors, NO, and prostanoids. They also participate in inducing endothelial adhesion molecules, which are crucial for leukocyte adhesion to the surface of endothelial cells. Thus, proinflammatory cytokines induce inflammation, tissue damage, and dysfunction, while anti-inflammatory factors block this process or suppress the intensity of the inflammatory response (42).

TNF-α is a proinflammatory cytokine produced by macrophages, natural killer cells, or T cells. It acts as an inflammatory marker closely related to diabetes, which is linked to metabolic disorders, including obesity and insulin resistance (43). Diabetes causes damage to a variety of tissues associated with this cytokine. For example, in a mouse model of type 2 diabetes, the interaction between TNF-α and IL-6 led to cardiac endothelial dysfunction (44); TNF-α participates in the recruitment of monocytes and macrophages, reduces the glomerular filtration rate through hemodynamic changes, and promotes the progression of diabetic nephritis (45). TNF-α also plays an important role in diabetic retinopathy. Elevated concentrations of TNF-α have been reported in the serum, vitreous, and aqueous humor of patients with DR (46, 47), while the level of TNF-α in the serum is positively correlated with the severity of the disease (48). It can increase the expression levels of endothelial nitric oxide synthase gene and intercellular cell adhesion molecule-1 (ICAM-1) and activate nuclear actor kappa B (NF-κB).These molecules all play a role in promoting the inflammatory response in DR. Additionally, it has been reported that TNF-α inhibitors reduce the inflammatory response in DR (49). However, TNF-α induces apoptosis, thus disrupting the normal function of the blood vessel wall and affecting the vascular permeability of the retina. TNF-α, which is released from Müller cells, causes apoptosis of retinal pigment epithelial cells by activating the EGFR/p38/NF-κB/p62 pathway. In diabetic mice, blockage of the TNF-a/EGFR axis relieves blood-retina barrier breakdown (50). Moreover, TNF-α causes the loss of retinal microvascular cells and promotes DR progression (51). In general, TNF-α participates in the inflammatory response, neovascularization and vascular reactivity.

IL-17A is a proinflammatory factor produced primarily by T cells. IL-17A knockout reduces the levels of TNF-a, IFN-c and IL-1b in Akita mice, suggesting that IL-17A is strongly associated with proinflammatory cytokine-driven inflammatory responses in diabetes progression (52). IL-17A plays a major role in increasing the intensity of retinal inflammation, oxidative stress, and vascular permeability in retinal disease (53). Blocking IL-17A alleviates diabetic retinopathy in rodents (54). In *in vitro* culture, retinal Müller cells display elevated expression of IL-17A and its receptor IL-17RA, along with increased secretion of IL-17A under hyperglycemic conditions. Meanwhile, IL-17A induced apoptosis of Müller cells through the Act1 pathway (55). Diabetic mice with IL-17A knockout display reduced retinal microvascular damage, retinal Müller cell abnormalities, and retinal ganglion cell apoptosis, which suggests that IL-7A is positively involved in DR pathophysiology (56). Furthermore, through IL-17A/IL-17R to the Act1/FADD signaling cascade, IL-17A causes degeneration of retinal capillaries and induces apoptosis of retinal endothelial cells (52). IL-17A acts as a proinflammatory cytokine that is primarily involved in retinal cell apoptosis and is expected to be a potential clinical target for the treatment of diabetic retinopathy in the future.

IL-1β is a multifunctional cytokine that promotes inflammation. It is rarely present in the cells of healthy individuals. Some cytokines, such as TNF-α, IL-18, and IL-1, including IL-1β itself, induce the production of IL-1β (57). Unlike TNF-α-induced insulin resistance, IL-1β has a direct killing effect on islet β cells (58). The findings are that IL-1β released by macrophages after a meal can synergize with insulin to activate the inflammasome, which can promote inflammation (59). In addition, IL-1β is a major trigger of the neuroinflammatory cascade (60). IL-1β was found to be increased in the retina of rats with diabetes as well as in the serum of proliferative diabetic retinopathy patients (61, 62). Studies have shown that the inflammatory status of DR is associated with a decreased degree of tyrosine nitrosylation of IL-1β in the vitreous (63). IL-1β damages retinal capillary endothelial cells by activating NF-κB and increasing oxidative stress, which can mediate mitochondrial damage, and it accelerates this damaging process in the case of hyperglycemia (64–66). Many studies have reported the effects of blocking IL-1β: pituitary adenylate cyclase-activating peptide administration reduces levels of IL-1β in rats with DR, thus protecting retinal tissue (67); blockade of IL-1β restores islet beta cell function over a short period or even allows some islet beta cells to regenerate (57); IL-1 blockers are effective in many autoinflammatory syndromes (68), suggesting their anti-inflammatory effects; and IL-1 receptor antagonists are effective in treating many eye diseases, such as uveitis and scleritis (69). These findings may start a new avenue for the treatment of DR.

IL-10 is an anti-inflammatory cytokine that plays a protective role in DR progression. Decreased IL-10 levels led to accelerated DR development in retinas of CX3CR1-deficient mice model (70). IL-10 improves the formation of subretinal fibrosis under the induction of exogenous HSP70, which helps avoid severe vision loss (71). While IL-10 acts as an anti-inflammatory factor, several studies have reported increased levels of IL-10 in the aqueous humor and vitreous of DR patients (72, 73). In addition, studies have reported that IL-1β is inversely correlated with IL-10 in the healthy population; however, when diabetes mellitus occurs, the balance between anti-inflammatory IL-10 and proinflammatory IL-1β is broken (72). One possible reason for the increase in IL-10 levels is that when inflammation occurs, IL-10 will display higher secretory activity to offset the rise in proinflammatory cytokine levels and prevent the development of inflammation.

IL-6 acts as a multifunctional inflammatory factor whose role is associated with immunomodulation, increased vascular permeability, and stimulation of angiogenesis (74). Higher concentrations of IL-6 were found in aqueous humor and serum in DR patients (75). IL-6 plays a role through classic and trans-signaling. Classic signaling exerts an anti-inflammatory effect and has regenerative activities, while trans-signaling is thought to be associated with proinflammatory activities (76). It has been discovered that inhibiting IL-6 trans-signaling lessens the oxidative damage that diabetes mellitus causes to the retina (77) and helps to minimize vascular inflammation and endothelial barrier issues (78). Following the activation of the IL-6 trans signaling pathway, the expression of adhesion molecules such as ICAM-1, VCAM-1 and selectins is increased (79). By rearranging actin filaments and altering the morphology of endothelial cells, IL-6 improves the permeability of endothelial cells *in vitro* (74). In addition, IL-6 stimulates the Jak/STAT3 pathway in the eyes, thus inducing apoptosis by the downstream receptor NO (80). Another important downstream effector of the STAT3 pathway is vascular endothelial growth factor (VEGF). It mediates pathological angiogenesis and increased vascular permeability. IL-6 can support angiogenesis by inducing VEGF indirectly in the state of DR (81). Inhibition of IL-6 and selective inhibition of IL-6 trans-signaling have entered clinical trials for treating a variety of inflammatory diseases and are expected to be a therapeutic target for DR (79).

### Chemokines

Chemokines are small heparin-binding proteins that can induce circulating leukocytes to move to inflammation or injury sites. According to their different structures and functions, chemokines segregate into four families, namely, CC chemokines, CXC chemokines, CX3C chemokines, and XC chemokines. The binding of chemokines to receptors activates the cascade of signals, which eventually leads to the rearrangement, shape change, and cell movement of actin (82).

MCP-1, which belongs to the CC family of chemokines, participates in the progression of vascular inflammation in DR and acts as a powerful chemokine for recruiting monocytes and macrophages (83). In the hyperglycemic state, MCP-1 is upregulated after NF-B activation, and diabetes patients may produce a significant amount of MCP-1 from Müller cells into the vitreous cavity and anterior chamber (84). By attracting monocytes, higher levels of MCP-1 in the diabetic retina affect the blood-retinal barrier and the permeability of retinal blood vessels (85). Retinal vascular ischemia is brought on by capillary blockage brought on by recruited leukocytes and macrophages adhering more strongly to the vascular endothelium (86). MCP-1 is one of the dominant causes of blindness in patients with DR. MCP-1 also exerts angiogenesis by inducing VEGF and activating RhoA (87). In addition, MCP-1 induces the activation of microglia, which release inflammatory factors, leading to the breakdown of optic vessels and damage to retinal neurons (88). Higher concentrations of MCP-1 have been found in vitreous samples of patients with DR (89); although MCP-1 primarily has an indirect impact on the progression of the illness, its importance cannot be understated.

C-X-C motif chemokine 12 (CXCL12), also known as stromal cell-derived factor 1 (SDF-1), is intimately linked to the development of type 2 diabetes and associated consequences. Patients with type 2 diabetes mellitus have increased serum levels of SDF-1, according to reports (90). Inhibition of SDF-1 in mice with diabetic nephritis reduces the severity of glomerular sclerosis and prevents proteinuria (91). Higher levels of SDF-1 have also been found in the vitreous of patients with DR, suggesting its pathogenic effect on eye lesions (92). SDF-1 promotes a firm adhesion of endothelial cells to the endothelium of the vasculature by increasing the expression of VCAM on endothelial cells; it also promotes the migration and homing of endothelial progenitor cells (93). It also acts as angiogenesis (94) with higher levels in ischemic and hypoxic retinas. It recruits endothelial progenitor cells to ischemic regions and synergize with VEGF and its receptor CXCR4 to participate in angiogenesis events (95, 96), which is one of the primary reasons why DR patients become blind. However, recent studies have reported that the SCF-1/CXCR4 pathway may improve DR by increasing cell activity, and further clinical studies are still needed to confirm this hypothesis (97).

Monokine induced by interferon-γ (MIG) is a CXC chemokine expressed in multiple cell types exposed to interferon-γ. It is usually connected to the Th1 response and directs the migration of activated lymphocytes, with an activity that inhibits angiogenesis (98). The levels of MIG increase in the vitreous of DR patients and are significantly related to VEGF (99). Compared to inactive proliferative diabetic retinopathy patients, the levels of MIG are significantly elevated in proliferative diabetic retinopathy patients with active neovascularization (100). The mechanism of action of MIG in DR has not yet been elucidated, and some studies believe that there is a positive regulatory feedback loop between MIG and VEGF, which may facilitate a regulatory angiostatic function (100). Another hypothesis is that MIG plays a role in the chemotaxis of leukocytes, rather than as an angiogenic inhibitor (74). MIG is known to have higher expression in other inflammatory diseases. MIG blockade has been described as a prospective therapeutic target for Crohn’s disease, and serum MIG levels represent the activity of the disease (101); in patients with rheumatoid arthritis, the expression of MIG is observed in the serum, synovial fluid, and synovial tissues (102). The inhibition of MIG in inflammatory diseases may present a potential therapeutic target for DR, but the feasibility remains to be checked.

IL-8 is the most well-known CXC chemokine, which has powerful proinflammatory properties resulting in its strict regulation, with low or no expression in normal tissues. Activated macrophages and monocytes release IL-8, which encourages the directed migration of basophils, neutrophils, and T cells. The eye effects of IL-8 vary depending on the site of action and the source of production, and one of its surprising effects is that it shows angiogenic activity in any part of the eye (103). Through angiogenesis and the proinflammatory response, IL-8 actively contributes to DR (104). Patients with proliferative diabetic retinopathy have significantly higher vitreous and aqueous fluid levels of IL-8 (75), which are linked to a greater amount of large-vessel gliotic obliteration in these patients (105). The increase in IL-8 in patients with poor visual prognosis after vitrectomy may damage the retina by recruiting ischemic inflammatory cells (104). Endothelial cell proliferation and inhibition of apoptosis can both be directly induced by IL-8 (106). In response to hypoxia, periretinal cells, ciliary epithelium, and glial cells release VEGF and/or IL-8, which stimulates the proliferation of endothelial cells and results in intraocular neovascularization (103). Diabetes mellitus can lead to retinopathy and hypoxia, while activation of NF-κB under hypoxia regulation increases the expression of IL-8 mRNA (107), which worsens retinopathy. Overall, elevated levels of IL-8 promote DR progression.

Fractalkine, also known as CX3CL1, is a CX3C chemokine that interacts with the specific CX3CR1 receptor on peripheral leukocytes such as microglia (108). Fractalkine has angiogenic activity both *in vitro* and *in vivo* and is substantially expressed in the vitreous of patients with proliferative diabetic retinopathy, suggesting that it may be a key factor in the progression of the disease (109). However, it has been shown recently that the absence of CX3CR1 in the DR mouse model of systemic inflammation leads to substantial perivascular clustering of proliferating microglia in regions of fibrinogen extravasation and increases the level of the proinflammatory factor IL-1β (110). Additionally, retinal ganglion cell layer neuronal cell counts in CX3CR1 deletion diabetic mice were lower, whereas microglial cell counts were higher and the microglia were more active (111). Microglia are resident monocytes in the retina. Activated microglia in DR patients release various proinflammatory mediators, including cytokines, chemokines, glutamate, and caspases, and enhance the expansion and migration of these mediators. These changes cause damage to retinal neurons, leading to blindness in patients with DR (112). While fractalkine activates the Nrf2 pathway and inhibits the NF-B pathway to deactivate microglia, this reduces the production of ROS and proinflammatory cytokines (113). In summary, the fractalkine/CX3CR1 signaling pathway plays a protective role in the diabetic retina.

### Adhesion molecules

White blood cell adherence to the microvasculature is one of the early events of diabetic retinal inflammation. Leukocyte adhesion accelerates the loss of endothelial cells and disrupts the blood-retinal barrier by releasing inflammatory cytokines, growth cytokines and vascular permeability factors (114). High expression of cell adhesion molecules promotes the effect of leukocyte adhesion.

Interccellular adhesion molecule 1 (ICAM-1), which is increased in disorders such uveitis, diabetes, and age-related macular degeneration, is crucial for the migration of white blood cells (115). It is also the main adhesion molecule involved in DR. Several studies have reported high expression of ICAM-1 in the vitreous of DR patients (116–118). ICAM-1, which binds to integrins on leukocytes, is induced by TNF-α. It can regulate the adherence and migration of leukocytes, causing retinal leukostasis (119). By interacting with a number of cytokines to breakdown the blood-retinal barrier, ICAM mediates the migration of white blood cells. Serum sICAM-1, which improves the adhesion between white blood cells and the vascular endothelium, is released from the outer segment of ICAM-1 (120).

Vascular cell adhesion molecule-1 (VCAM-1), which binds to integrin, is expressed on endothelial cells (119). It is overexpressed in the diabetic fiber vascular membrane (118) and is raised in conditions of hyperglycemia or hyperlipidemia (121). TNF-α regulates the expression of VCAM-1 but has a dual effect. It reduces the level of VCAM-1 under basal conditions but promotes retinal endothelial activation in response to diabetes (121). The antiangiogenic drug conbercept has a significant inhibitory effect on VCAM-1 expression in the retina of mice with proliferative diabetic retinopathy, which prevents retinal endothelial cell proliferation (122). Although it is currently known that VCAM-1 is related to endothelial function, its function in DR is not well understood.

Elevated levels of selectin induce the aggregation of leukocytes into the endothelial wall and then lead to retinal leukostasis. It can be divided into three classes depending on the expressed cell type. L-selectin, which is primarily responsible for moving leukocytes to inflamed tissues, is expressed on circulating leukocytes (123). Only endothelial cells express E-selectin, and its expression is increased when cytokines or ROS are present (124). According to reports (125), the presence of retinopathy is associated with higher levels of soluble E-selectin. P-Selectin (126) is expressed in endothelial cells as well as platelets, and it is expressed more frequently in DR patients. This process involves endothelial barrier alteration (48).

Integrins are a large group of membrane-binding proteins with 18 α subunits and 8 β subunits in vertebrates that can form different heterodimers. The function of integrins is to allow white blood cells to pass through the blood vessel walls, and they are receptors for cell adhesion during DR development. On the membrane of leukocytes, β2 integrin binds to ICAM-1, while α4β1 and α4β7 bind to VCAM-1 on endothelial cells (127, 128). Integrins make a difference in the progression of eye diseases. As an example, integrins αVβ1 and α3β1 are involved in the infectious process of corneal tissue in allergic eye disease (129). The pathogenic process underlying glaucoma is significantly influenced by αVβ3 integrin (130, 131). For DR, it has been noted that patients with proliferative diabetic retinopathy have higher amounts of αvβ3-, α5- and αvβ5-integrins in their fibrovascular membranes (132). In the human eye, angiogenesis can be induced through two pathways of integrins: first, αvβ3 mediates angiogenesis in models of corneal or chorioallantoic angiogenesis involving TNF- and basic fibroblast growth factor; second, VEGF or transforming growth factor is mostly mediated by αvβ5 during angiogenesis (133). Additionally, diabetic retinopathy in an animal model is induced by α4 integrin/CD49d, which also facilitates leukocyte adhesion. By inhibiting the NF-B pathway, blockade of α4 integrin/CD49d can reduce vascular leakage and leukocyte adhesions (134). As mentioned before, β2 integrin binds to ICAM-1 to participate in adhesion to the blood vessel wall, induces downstream leukocyte activation and promotes inflammation. After the activation of white blood cells, it reacts to the increase in the expression of β2 integrin (135). In conclusion, integrins promote the function of other adhering molecules and cause angiogenesis in the development of DR.

Although DR is considered a microvascular disease, there is increasing evidence that a low-grade inflammatory state of the retina is an early manifestation of DR. Increased levels of multiple cytokines, chemokines and adhesion molecules are found in eye tissue of DR patients. Proinflammatory cytokines mediate the downstream inflammatory signaling pathways, directly promote the progress of DR. Chemokines recruit leukocytes that secrete cytokines to inflammatory sites, making cytokines in functional spatial position; adhesion molecules then bind leukocytes to inflammatory sites, prolonging the action time of functional cells. Chemokines and adhesion molecules help cytokines to enhance inflammation indirectly. The long-term inflammatory state makes the blood-retinal barrier damage, retinal cell apoptosis, and ultimately leads to DR patients’ visual loss.

## Therapies targeting inflammation in DR

Over the past decade, advances in drugs and therapies have improved the prevention and treatment of patients with DR. The DR preferred practice pattern recommended that regular follow-up and necessary and appropriate retinal photocoagulation and vitrectomy can prevent severe visual loss in 90% of patients (136). Retinal laser photocoagulation is an important method for the treatment of DR and can be divided into panretinal photocoagulation (PRP) and macular laser treatment. Frequent laser treatment and vitreous surgery can have adverse effects on patients, such as apoptosis of retinal pigment epithelium and other retinal cell types and reduced vision (126).

Optical Coherence Tomography (OCT) is a non-invasive fundus imaging device, which has great clinical significance in the screening, diagnosis, follow-up, and evaluation of therapeutic effect of DR. Serous retinal detachment (SRD) and high reflectivity points (HRDs) on OCT may be related to the anti-inflammatory effect of DME. One of the early events in the pathogenesis of DME is microglia activation. The microglia is an intrinsic macrophage that sits around blood vessels in the inner layer of the retina and is involved in the maintenance of BRB. It is the sentinel cell of the retina. The activated microglia in OCT are seen as high reflectivity points between the retinal layers. OCT showed an increase in interlaminar hyperreflectivity in all diabetic patients, especially in diabetic retinopathy patients (137). The more the number of high reflex points, the worse the effect of anti-VEGF therapy, and the better the effect of hormone therapy (138). SD-OCT can help to predict the visual prognosis of DME patients. Serous retinal detachment is more common in more severe DME, and about 30% of DME is associated with serous retinal detachment (139). Serous retinal detachment suggests an inflammatory factor. Hormones are more effective against serous retinal detachment than anti-VEGF drugs (140).

Although clinicians can use many therapeutic strategies to treat DR, no treatment can completely attenuate clinical progression to reverse retinal damage. More current strategies for the treatment of DR aim to improve therapeutic efficacy, as well as noninvasive or alternative delivery mechanisms that provide a longer duration of action. **Table 1** summarizes existing clinical trials that have been completed to treat DR.

**Table 1:** Completed clinical trials of drug treatments in patients with diabetic retinopathy.

Intervention	Pathways	Completed clinical trials
Metabolic control (Hypoglycemic Agents, Fenofibrate, Aliskiren)	Insulin signaling, RAAS	NCT00542178 (141), NCT00768040, NCT00252733 (142), NCT00252720 (142)
Ruboxistaurin	PKCβ signaling	NCT00266695, NCT00090519, NCT00604383 (143)
Bevacizumab, Ranibizumab, Aflibercept, Conbercept, Pegaptanib Sodium	VEGF signaling	NCT01100307, NCT01270542, NCT01908816, NCT01363440, NCT00682539, NCT01661946, NCT00548197, NCT02834663, NCT01988246, NCT01594281, NCT00996437, NCT02366468, NCT02816710, NCT01854593, NCT02718326 (144), NCT01069341, NCT02858076, NCT01805297, NCT00606138, NCT00445003, NCT00745498, NCT01189461, NCT03126786, NCT02949024, NCT03917472, NCT02863354, NCT05414149
Intravitreal steroids(Triamcinolone acetonide, Dexamethasone); NSAID; Antibiotics(doxycycline monohydrate); Immunosuppressants	Inflammation	NCT01853072 (145), NCT00444600, NCT01571232, NCT00917553, NCT00511875, NCT00711490, NCT00782717, NCT01872611 (145), NCT00105404, NCT00367133, NCT02443012, NCT03608839, NCT00229931, NCT00369486, NCT01892163, NCT02511067, NCT02842541 (146), NCT01609881
Emixustat Hydrochloride	Visual cycle isomerohydrolase	NCT02753400 (147), NCT00412451

### Control of metabolic disorders

The fluctuation of blood glucose and hypoglycemia can aggravate ocular fundus changes, and intensive glycemic control can prevent and delay the occurrence and progression of DR. The Diabetes Control and Complications Trial (DCCT) proved that intensive treatment reduced the risks of DR (148). The burden of diabetic retinopathy may be lessened by renin-angiotensin system (RAS) blockers. The Diabetes Retinopathy Candesartan Trials (DIRECT) Programme (NCT00252733, NCT00252720) evaluated whether candesartan might slow the development and progression of retinopathy in T1D patients (142). Although the development of retinopathy is unaffected, candesartan may lower the incidence of retinopathy. In transgenic (mRen-2)27 rats, aliskiren, as a renin inhibitor, reduced intercellular adhesion molecule-1 to control levels and provided the same protection as ACE inhibition against proliferative neovascularization of NPDR and oxygen-induced retinopathy (149). RAS blockers are recommended as the first choice for diabetic patients with hypertension, but RAS blockers are not recommended for the prevention of retinopathy in normotensive diabetic patients. The Action to Control Cardiovascular Risk in Diabetes (ACCORD) study (NCT00542178) has investigated whether intensive glycemic control, combined therapy for dyslipidemia, and intensive blood pressure control limit diabetic retinopathy progression in patients with type 2 diabetes (141). This 10-year duration study found that intensive glucose control and intensive combination therapy for dyslipidaemia reduced the rate of diabetic retinopathy progression.

### Anti-angiogenic therapies

VEGF is an important factor involved in the pathophysiological process of DR and DME. Hypoxia and hyperglycemia may lead to the upregulation of VEGF, which may lead to leakage and vascular proliferation. There is a large amount of evidence showing the efficacy of anti-VEGF therapy in DME. Currently, bevacizumab, aflibercept, conbercept, and pegaptanib sodium have been studied in clinical studies as anti-VEGF medications for the treatment of DR (**Table 1**). Several large randomized trials have expanded on the initial findings of the Diabetic Retinopathy Clinical Research Network (DRCRnet) to show that other VEGF agents (bevacizumab, ranibizumab, and aflibercept) are also superior to laser therapy (150). Pegaptanib sodium, an RNA aptamer targeting VEGF-165, was approved by the Food and Drug Administration (FDA) in 2006 and is the first VEGFA inhibitor in ophthalmology. Pegaptanib sodium can be used in DME and appears to be well tolerated with evidence of efficacy, but vision is still declining in most patients; now, it is rarely used in clinics (151).

In 2004, the FDA authorized bevacizumab as the first antiangiogenic medication for use in the first-line treatment of metastatic colorectal cancer (152). It is a recombinant human IgG-1 monoclonal antibody against VEGF that prevents VEGF from binding to VEGFR by binding to VEGF, and inhibition of endothelial proliferation and activation leads to antiangiogenic and antitumour effects. In large clinical trials, ranibizumab has been shown to be effective and safe in DR treatment, improving DR severity in both NPDR and PDR (153). The researchers investigated the relative effectiveness and safety of glass injections of aflibercept, bevacizumab, and ranibizumab in the treatment of DME. In patients with central involvement of DME, it was discovered that vitreous injections of aflibercept, bevacizumab, and ranibizumab enhanced visual acuity and decreased retinal thickness, although the proportionate benefit depended on baseline eyesight (154).. At a lower initial level of vision, aflibercept was more effective at improving vision. In the DRCR Retina Network trial, which involved moderate vision loss due to DME, the team did not find that over a two-year period, there was a significant difference in visual outcome between aflibercept monotherapy and bevacizumab treatment, and in the event of a poor response, aflibercept may be preferred (155).

Conbercept is the first new biological class I drug with independent intellectual property rights in China and has been given the World Health Organization’s (WHO) international generic name. As a new generation of anti-VEGF fusion proteins, conbercept can inhibit choroidal angiogenesis and reduce the leakage of new blood vessels (156). At the time of the phase III trial, subjects were unable to schedule treatment every 8 weeks or 12 weeks because of the COVID-19 outbreak, and several studies are still recruiting patients. However, the limitations and adverse effects of anti-VEGF therapy have also received much attention. Because anti-VEGF drugs have a short half-life, they need to be injected monthly or every two months to ensure efficacy. In patients receiving anti-VEGF, conjunctival hemorrhage, eye discomfort, cataract, vitreous detachment, vitreous floaters, and elevated intraocular pressure were the most frequently reported side effects (5%) (157). Endophthalmitis and retinal detachment may occur after intravitreal injection. There are also studies to improve injection methods, such as the Port Delivery System (PDS), which is a permanent, repeatable, and small-size eye implant that can deliver custom-formulated ranibizumab over several months. There is potential to reduce the treatment burden of frequent eye injections. In phase 2 trials, PDS was generally well tolerated, reducing the burden of therapy for individuals with neovascular age-related macular degeneration (nAMD) while maintaining visual acuity (158).

### Anti-inflammatory therapies

Several studies have demonstrated that inflammation is involved in the pathogenesis of DR. Therefore, anti-inflammation is not only an effective supplement to DR therapy but also an important treatment for some patients who are ineffective or resistant to anti-VEGF therapy. Before the use of VEGF drugs, intravitreal glucocorticoid therapy was popular among treating physicians. Steroids reduce neutrophil migration, restrict access to inflammatory sites, and reduce cytokine production (159, 160). In the nonproliferative phase of DR, DME is the main cause of visual impairment. Dexamethasone vitreous implants have been effective in treating DME indications since the FDA was approved in 2014 (161). The Chinese phase III study of dexamethasone intravitreal implants (DEX-I) for DME patients enrolled 284 Asian patients from 18 centers, and the visual acuity, macular edema thickness, and leakage area of patients treated with DEX-I were better than those treated with laser photocoagulation. Although glucocorticoids such as triamcinolone acetonide and dexamethasone implants have also been shown to reduce retinal thickening and improve vision, the use of glucocorticoids for intravitreal treatment increases the risk of cataract surgery and can lead to elevated intraocular pressure and glaucoma (162). Therefore, the complications of high intraocular pressure and cataract formation should be considered in vitreous glucocorticoids.

Non-steroidal anti-inflammatory drugs (NSAIDs) can reduce damage to the DR retina by inhibiting the expression of inflammatory factors and nonsteroidal anti-inflammatory drug mediators to control the retinal inflammatory reaction. Several studies assessing the effect of localized NSAIDs on diabetic retinopathy have reported improvements in fovea thickness and vision at approximately four to six months (163). Nepafenac ophthalmic (Nevanac, Alcon), the first eye NSAIDS product authorized by the FDA in 2005, is used to relieve pain and inflammation associated with cataract surgery. After being given to the eye, nepafenac can pass through the cornea quickly and transform into aminophenic acid under the influence of ocular tissue hydrolase, which can quickly reach the target location to suppress the activation of caspase-3 and -6 in the retina (164, 165). Nepafenac 0.3% showed superior clinical outcomes than vehicles in two prospective, randomized, multicenter, double-masked, phase 3 trials in patients with DR, with better BCVA after cataract surgery and no unexpected adverse events (145). NSAIDS has definite clinical efficacy and high safety. The adverse reactions disappear automatically after discontinuation of NSAIDS and do not affect the efficacy. However, NSAIDS can cause corneal melting and even perforation, which should be given more attention. The use of NSAIDS in patients with autoimmune diseases, eye diseases, and other diseases should be done with caution.

IL-6 has been investigated as a viable target for anti-inflammatory treatment of DR since it is one of the most significant proinflammatory cytokines in the vitreous of patients with DR. Some clinical trials have led to the development of antibodies against IL-6 (EBI-031) and the IL-6 receptor (tocilizumab). The safety, tolerability, and effectiveness of tocilizumab, a recombinant humanized anti-human interleukin-6 (IL-6) receptor monoclonal antibody, have been examined in clinical studies in eyes with DME (NCT02511067). Clinical research (NCT02842541) is evaluating the safety, tolerability, immunogenicity, and pharmacokinetics of up to three dosage levels of EBI-031 administered intravitreally to participants with diabetic macular edema (NCT02842541). The interventional study found that ketorolac (Acuvail^®^) concentrations (0.45%) significantly reduced the levels of aqueous IL-8, vitreous IL-8, and platelet-derived growth factor (PDGF) AA in 20 eyes from 20 patients, which suggests that it may induce significant inhibition of inflammatory cytokines involved in the pathogenesis of DR (146).

Vascular adhesion protein-1 (VAP-1) regulates leukocyte adhesion and has semicarbazide-sensitive amine oxidase (SSAO) functions to affect oxidase activity, catalyzing oxidative deamination to produce hydrogen peroxide and aldehydes, leading to the production of AGEs and ALEs (166). PXS-4728A is mainly used in the treatment of cardiometabolic diseases. In acute lung inflammation, PXS-4728A, as a recently reported SSAO inhibitor, was used in the treatment of cardiometabolic diseases. In acute lung inflammation, PXS-4728A reduced CXCL1/KC-induced leukocyte rolling and adhesion (167). In arteriosclerosis, PXS-4728A reduced oxidative stress and the expression of adhesion molecules, chemoattractant proteins and proinflammatory cytokines in the aorta, and it also inhibited the adhesion and migration of monocytes in human umbilical vein endothelial cells (168). Currently, a clinical study that tested the safety and efficacy of BI 1467335 (PXS-4728A) has shown some improvement in patients with DR, with no increase in adverse events (NCT03238963).

Montelukast, a leukotriene receptor antagonist, revealed a protective impact on vision in a streptozotocin-induced diabetes mouse model by inhibiting diabetes-induced capillary and neuronal degeneration (169). Drug blockade of the leukotriene pathway holds the potential for new therapies to prevent or slow diabetic retinopathy development. At present, there is no clinical evaluation in the treatment of DR.

Connexin43 translucency plays a role in the pathogenesis of chronic inflammatory diseases, including activation of the inflammasome pathway, and sanguinarine blockade has been shown to alleviate vascular leakage and inflammation (170, 171). Hyperglycemia and inflammation increased Connexin43 expression in both the Akimba (DR) mouse retinas and the donor retina with confirmed DR (172). Tonabersat, a connexin hemichannel blocker, can inhibit NLRP3 and lysed Caspase-1 complex formation with hyperglycemia and cytokine activation while preventing the release of the proinflammatory cytokines IL-1β, VEGF, and IL-6 (173). Tonabersat reduced retinal inflammation by modulating the assembly of the inflammasome (NLRP3) through Connexin43 translucent blockade while preserving retinal photoreceptor function and restoring vascular integrity (174). Tonabersat has the potential to improve some functional outcomes in diabetic retinopathy and is a potential therapeutic agent.

AKST4290, an inhibitor of CC chemokine receptor-3 (CCR3), is a natural receptor for eotaxin. Wet age-related macular degeneration (wet AMD), as well as other neurological and immunological illnesses, is mostly a result of the pathophysiology of inflammation, immune cell recruitment, and neovascularization, which is regulated by CCR3 (175). The oral medication AKST4290, which works well in preventing eotaxin from attaching to its G-protein coupled receptor (GPCR) CCR3, increased the age-related macular degeneration BCVA score (176). The effectiveness of oral AKST4290 in patients with moderate to severe diabetic retinopathy (CAPRI) is currently being studied (NCT05038020).

### Other therapeutic agents

At present, there are a number of drugs being studied for the treatment of DR. First, Runcaciguat, which has been studied from diabetes-related diseases. Runcaciguat (BAY1101042), as a sGC activator, may be an expansive therapeutic option for the prevention of CKD associated with hypertension, diabetes, and obesity (177, 178), which is currently in a phase II clinical trial (NCT04722991).

To develop some visual mechanisms, a nonretinoic acid small molecule called emixustat hydrochloride inhibits RPE65, also known as retinol isomeric hydrolase, which is a 65 kDa protein found in the retina (179). In 23 PDR patients with or without DME, the effects of oral emixustat hydrochloride on proangiogenic and inflammatory cytokines (levels of IL-1, IL-6, IL-8, TGF-1, and VEGF) were assessed. VEGF levels were marginally lower in the emixustat hydrochloride group, although this preliminary investigation did not demonstrate a statistically significant difference in changes in aqueous humor cytokine levels between the emixustat hydrochloride group and the placebo group(NCT02753400) (147). OTT166 is a novel small-molecule-selective integrin inhibitor specifically designed by OcuTerra Therapeutics, Inc. to have the required physicochemical properties to reach the retina from eye drops (180). OTT166 is currently being given in a phase II clinical trial in diabetic retinopathy patients (NCT05409235).

In addition, fenofibrate is a peroxisome proliferator-activated receptor alpha activator (PPARα) involved in the regulation of lipid metabolism disorder, inflammation, oxidative stress, angiogenesis and apoptosis, which reduces the progression of DR (181). It was found that PPARα was downregulated in diabetic retinas, which may be partly due to the overexpression of microRNA-21 (miR-21). In db/db mice, knockdown of miR-21 prevented PPARα downregulation, alleviated microvascular damage, and improved neovascularization and inflammation of the retina (182).

## Concluding remarks and future perspectives

**Figure 3:**
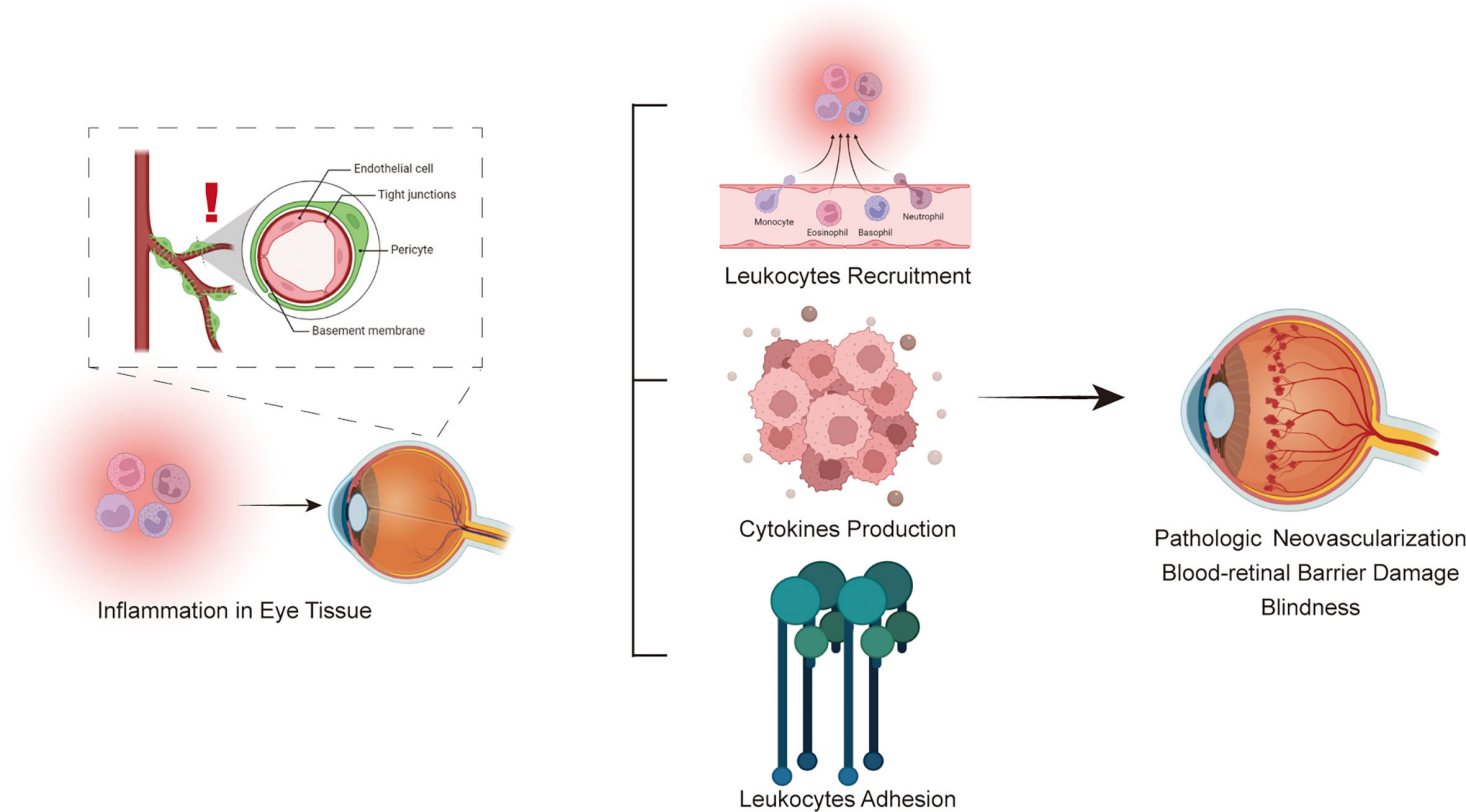
Schematic flowchart for the immune system involved in the pathophysiology of diabetic retinopathy.

Anti-inflammatory approaches targeting certain molecular markers might be viable treatment options for DR since inflammation is now recognized as a significant contributor to the onset and progression of DR. Cytokines, chemokines and adhesion molecules participate in the inflammatory process of diabetic retinopathy. Three types of molecules interact and are inseparable, forming a complex molecular network that promotes the pathological process of diabetic retinopathy(**Figure 3**). Cytokines are divided into proinflammatory and anti-inflammatory cytokines according to their function. When inflammation occurs, the levels of proinflammatory cytokines are upregulated, promoting inflammatory responses through a variety of pathways, such as retinal cell apoptosis and angiogenesis. Anti-inflammatory cytokines may also reach a higher level to counteract the protective effect of the inflammatory response. Chemokines, as the transport intermediary of cells, induce circulating leukocytes to reach inflammatory sites. Adhesion molecules help white blood cells adhere to inflammatory sites. Three types of molecules perform their own functions, while an increase in the level of one type of molecule also induces the expression of another type of molecule in an inflammatory state; for example, the cytokine TNF-α upregulates the level of the adhesion molecule VCAM-1. The complex pathological mechanism has not yet been elucidated and needs further study in the future. In the future, the immune inflammation mechanism of DR can be further studied to provide insight into the biological function of DR and related targeted therapies. Therefore, these findings imply the clinical importance of new therapies targeting inflammatory responses in the management of DR and facilitate the transfer of recent research findings from ‘bench to bedside’ in the future.

## Author contributions

Conceptualization; writing-original draft and editing: TY, YS. Writing-review and editing: SL. Writing-review and editing: JW, YW, XZ. All authors contributed to the article and approved the submitted version.
